# The Stressful Experience of Goal Orientations Under Frustration: Evidence Using Physiological Means

**DOI:** 10.3389/fpsyg.2022.823655

**Published:** 2022-04-13

**Authors:** Faye Antoniou, Ghadah S. Alkhadim

**Affiliations:** ^1^Department of Educational Sciences, National and Kapodistrian University of Athens, Athens, Greece; ^2^Department of Psychology, College of Arts, Taif University, Taif, Saudi Arabia

**Keywords:** goal orientations, stress, frustration, physiological analysis, experimental study, normative goals, outcome goals, ability goals

## Abstract

The purpose of the present study was to test the hypothesis that goal orientation is associated with divergent forms of emotional reactivity under frustration. Goal orientations were assessed using bifurcations of performance goals described earlier. Physiological stress levels were measured *via* a blood volume pulse analysis after individuals were subjected to a computerized Stroop task using a malfunctioning mouse to induce enhanced frustration. The results indicated that performance-avoidance goals were associated with the highest levels of emotional reactivity, with normative outcome goals being significantly more detrimental than ability goals. We concluded that the motivation to avoid failure or to outperform others is the most detrimental determinant of stress and needs to be avoided by all means. Instead, it is suggested that educators emphasize performance using personal best outcomes or by valuing engagement, deep processing and task completion.

## Introduction

Stress is one of the most significant obstacles to effective self-regulation. At high levels, stress depletes cognitive resources by pressuring the cognitive mechanism and misallocating them to irrelevant tasks and processes (e.g., worrying rather than problem-solving) (see Carr (2015) for a review). Furthermore, it has been linked to serious illnesses or even death (Abramson et al., 2002).

Among objective stress response measures, one of the most significant is the blood volume pulse (BVP), which assesses the amount of blood that passes through a photoplethysmographic (PPG) sensor (Peeper et al., 2007) in order to transfer nutrients and oxygen to organs and tissues. Excessive arousal, such as when fearful events are encountered, are associated with vasoconstriction (decrease in blood perfusion), whereas events related to positive affectivity are associated with vasodilation (Egloff et al., 2002). Needless to say, the former has been linked to poor physical (Taylor, 2010) and psychological health outcomes (Patron et al., 2012), and even death (Kleiger et al., 1987), and not only in the presence of stressful events; vasoconstriction is an index of aging as well, as it reflects poor vascular wall elasticity leading to high blood pressure and hypertension (Izzo and Shykoff, 2001). Thus, a BVP analysis provides an overall index of cardiovascular health (Asada et al., 2003), but can also be used for the temporal evaluation of stress.

The second salient indicator of the stress response is manifested by excessive movement. Several studies have suggested that increased electromyography (EMG) is causally associated with stress experiences (Rissén et al., 2002; Luijcks et al., 2014). For example, Lundberg et al. (1994) applied, similar to the present study, a color Stroop task to assess the effects of mental stress on muscle reactivity. Through employing 62 female participants, they showed that two mental stress tests significantly increased EMG activity. These results have been replicated in subsequent studies (Wahlström et al., 2003).

Stress may also signal cognitive impairment, as it may interfere with information processing in complex cognitive tasks. This concept of cognitive interference, defined by Sarason et al. (1996), refers to a flow of unwanted and disturbing thoughts that distract the person and interfere with everyday functioning. This observation is in light of resource allocation theory (Ellis and Ashbrook, 1988), which stated that irrelevant to a task thoughts overload the cognitive mechanism, thus reducing cognitive capacity (Papantoniou et al., 2012). For example, responses to the stress created by pressure to maximize one’s task performance include such cognitive interference, as shown, for example, in Beilock and Carr (2001), Beilock et al. (2004) two-part analysis of theories of choking under pressure. Enhanced pressure may lead to choking when specific conditions, termed “pressure operators” (Beilock and Carr, 2005; Hill and Shaw, 2013; Mesagno and Hill, 2013) are met. These operators can be *economic rewards* (e.g., grades), *social responsibility* (i.e., effect of one’s achievement on a team), *public scrutiny* (e.g., other people watching) and *evaluation* (e.g., judgments over one’s performance). Interestingly, operators 1, 3, and 4, are significant components of the operational definition of normative performance goals (i.e., a focus on grades, exposure to the public and social comparisons, and normative evaluations). Based on the work of Beilock and colleagues (Beilock et al., 2004; Beilock et al., 2006) pressure enhances feelings of worry and forms perceptions on the likelihood of success. In their view, these thoughts “compete for space in working memory that could otherwise be devoted to goal structures, task-control information, and task-relevant data….” (p. 9). Collectively, confirming these hypotheses in any given performance situation leads to adoption of a cognitive-interference-based account (called “distraction theory” in the literature on response to pressure) as task-irrelevant thoughts intrude and overwhelm the information processing mechanism, leading to performance decrements.

Several studies have suggested that the activation of different electroencephalogram (EEG) waves signals different levels of cognition, such as the alpha and beta waves evaluated in the present study. According to Kirschfeld (2005), the alpha wave represents the most conspicuous signal in human EEG. Based on his analysis, reduced alpha waves amplitudes are linked to enhanced processing of information and the tendency to “move forward” and emit motor activities. Increased attention and focus have a negative relationship with alpha waves; thus, they are not considered conducive to cognitive tasks. Beta waves, on the other hand, are present when a person is alert or attentive and actively thinking. They are most prominent under conditions of deep concentration and engagement with problem-solving activities.

### Goal Orientation and Emotional Regulation

Motivation is the guiding force in task engagement and execution. Grant and Dweck (2003) put forth an interesting conceptualization of motivation by extending previous achievement goal theory frameworks. Specifically, they maintained the well-established idea that learning, or mastery goals are grounded in intrinsic interest, task value, active engagement, and intrinsic motivation, and sought to elaborate on the functionality of different types of performance goals based instead on evaluation of performance outcomes rather than intrinsic interest and motivation. Grant and Dweck recommended that performance goals that involve others as a reference point [see also Dunning (2017)] should be differentiated from those that involve performance, but the reference point is internal to the person, such as when using self-referenced standards. Thus, they termed *normative outcome goals* the goals that target at validating one’s ability by employing interpersonal evaluative standards such as between-person comparisons. An example item that captures the essence of these goals, based on Grant and Dweck (2003) is “I tried to do better in my classes than other students.” Grant and Dweck (2003) distinguished normative performance goals from a self-referenced performance goal, namely ability-based goals (see also Butler (1992, 2006) for earlier conceptualizations). The content of these goals refers to demonstrating ability such as showing “how smart one is”, but in the absence of normative evaluative criteria. Martin (2006), and Martin and Elliot (2015) presented another type of self-referenced performance goals they termed as personal best which they defined as “specific, challenging, competitively self-referenced targets to which students strive to match or exceed a previous best.” (p.222). Based on Liem et al. (2012) personal best goals avoid the negative consequences of social comparisons by using past performance as a benchmark. *Performance-avoidance goals* have been consistently defined using a focus on avoiding negative outcomes, such as failure, and have been largely responsible for decrements in intrinsic motivation. Last, engagement goals have been referred to as teacher and classroom goals in that students’ active engagement is sought so that deep processing and task completion will be accomplished (see Table 1 for these different goal conceptualizations as defined for use in this study).

**TABLE 1:** Achievement goal constructs used in the present study.

Achievement goal orientations	Theoretical framework	Goal content
1. Normative outcome	Grant and Dweck (2003)	To demonstrate competence by outperforming others
2. Ability	Grant and Dweck (2003)	To demonstrate competence using self-referenced standards
3. Performance avoidance	Elliot and Church (1997).	To avoid demonstrating lack of ability through avoiding failure
4. Personal best	Martin (2006), Butler (1992)	To match and/or exceed previous best
5. Engagement goals	Fredricks et al. (2004), Reeve et al. (2004)	To maintain active engagement and effort toward task completion

### Goal of the Present Study

Ample research studies have linked achievement goals to adaptive and maladaptive patterns of responding. What is less known, however, is the emotional response associated with these conceptualizations (Pekrun et al., 2009), as well as their validation using objective means such as physiological responses. To address this, the present study was designed to assess indicators of the stress response of individuals as a function of goal orientations under an experimental manipulation that targeted at inducing frustration. It was expected that the emotional manifestations of these goals would become more prevalent as the experience of a piece of malfunctioning equipment would likely represent an obstacle to goal attainment. Thus, our choice for inducing a frustrating experience was so that goal orientations would be evaluated under pressure and under demanding and uncommon environmental demands (Carr, 2015). Such demands and pressures that cannot be overcome (i.e., maladaptive equipment) would likely energize physiological responses that mimic real life achievement situations, which is the proper setting to evaluate achievement goals. Previous laboratory experiments have been criticized on the grounds that induced pressures were of low magnitude with also low levels of nervousness and anxiety (e.g., Mesagno et al., 2011). In the present study, levels of frustration were high based on our rating scale system (see section 2.2). Such levels were potentially associated with experiences of “chocking under pressure” and disengagement due to a dysfunctional focus on avoiding imminent failure (Chib et al., 2012). Thus, the goals of the present study were to contrast the emotional experience from adopting different goal orientations using goal formulations from Butler (1992), Grant and Dweck (2003), Fredricks et al. (2004) and Martin (2006).

## Method

### Participants and Procedures

Fifty college students (16 male, 34 female) from education and psychology backgrounds responded to a call to participate in an experiment for extra credit in a public university. They were healthy freshmen and sophomore students, free from any physical, emotional, or mental disability. Upon entering the lab, they were briefly informed of the experimental procedures and were asked to sign a consent form. Subsequently, they were informed of the tasks and procedures involved and were allowed to ask questions pertaining to the experiment. Following the briefing, the research assistants placed the sensors from the Biotrace system on the participants and ensured that the system worked properly after collecting trial data. Initial measures included warm-up sessions, pre-baseline evaluations to ensure the stability of physiological responses, and baseline assessments followed by experimental manipulations. A one-minute relaxation interval was applied between each experimental condition.

### Experimental Design and Manipulations

**FIGURE 1:**
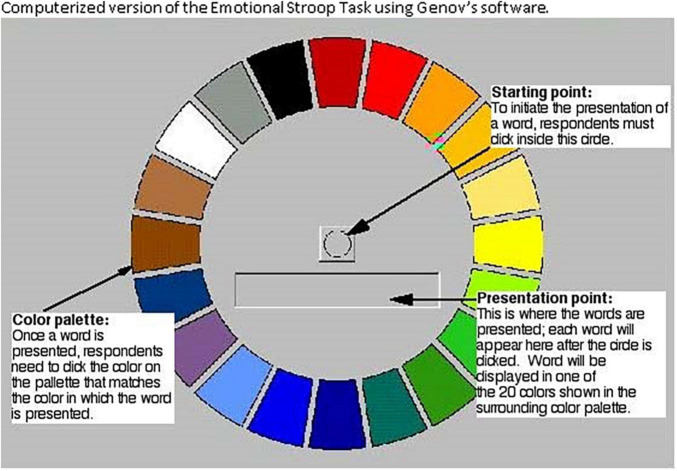
Computerlzed version of the Emotional Stroop Task using Genov’s software.

The experiment involved a computerized adaptation to the classic Stroop task in which stimuli appeared on a monitor and reaction times were assessed using a mouse (Genov et al., 2002) (see Figure 1 for test palette). Frustration was implemented using a severely malfunctioning mouse on the Stroop task platform and is in light of past studies that also implemented the Stroop paradigm to experimentally induce stress (Reinhardt et al., 2012). To address the potential caveat that frustration was under experimental control, we tested the validity of the manipulation by administering a 3-item questionnaire at the end of a session that collected information about the degree to which the malfunctioning mouse induced frustration. The three validity items evaluated were: (a) how annoyed participants were about the malfunctioning mouse, (b) how disappointed they were, and (c) whether they thought about quitting due to the malfunctioning equipment. The content of these items was selected in light of the definition of frustration using Oxford’s dictionary in which the closest synonym words were: annoyance, anger, irritation, bitterness, disappointment, dissatisfaction, discontent, and aggravation. Based on the Cambridge dictionary, frustration was defined as “the feeling of being annoyed or less confident because you cannot achieve what you want”. The scaling system involved five options: not at all (1), a little (2), somewhat (3), a lot (4), and very much so (5). Results indicated that the mean frustration levels were 3.87 (out of 5), suggesting that participants were approximately “a lot” annoyed by the malfunctioning equipment, verifying the induction of a frustrating experience.

### Goal Experimental Conditions

Participants were randomly assigned to conditions to avoid ordering effects using a Latin square design. This randomization ensured that no two participants obtain the same order of experimental conditions. The content of the directions followed the conceptualizations of Grant and Dweck (2003) and Martin and Elliot (2015) on these goals (see Table 1). Given the intrinsic nature of mastery goals, they were excluded from the current manipulation. The rationale behind this decision was that mastery goals are intrinsic in nature and cannot be induced in the absence of intrinsic interest, attitudes toward learning and task value. The following conditions were implemented:

#### Normative Outcome Goals

They emphasized normative evaluative criteria and participants were given the following instructions: “With this task, we would like you to try and outperform everybody else.” This direction was repeated every 15 s.

#### Ability Goals

They focused on demonstrating ability using absolute evaluative criteria, such as demonstrating how smart one is. Specifically, participants were instructed to: “show me how smart you are” without any reference to external comparisons. As with the outcome goals, a repeat of the directions was provided every 15 s.

#### Performance-Avoidance Goals

They were targeted to induce fear of failure. Specifically, participants were told to “try not to fail,” and, “not to be among the worst performers in this task.” These instructions were repeated every 15 s.

#### Personal Best Goals

They targeted high achievement with no emphasis on social comparisons. Specifically, participants were directed to “try to do the best you can,” and, “try to do even better than the previous condition.” The directions were repeated every 15 s.

#### Engagement Goals

They targeted at enhancing active engagement with no reference to intra-individual or inter-individual standards. Participants were told: “I like how you work, please continue working like that.” This direction was repeated every 15 s.

To ensure validity of the goal manipulations, participants were asked to confirm the assigned goal. Four participants failed to reproduce the manipulated goal and were excluded from the study.

### Measures

As for indicators of a frustrating experience, we selected blood volume pulse, facial muscular activity, and indices of alertness and optimal brain functioning for cognitive tasks. These are discussed next.

#### Blood Volume Pulse

It was assessed using data from an ECG analysis with sensors placed on the Cz location, left ear, and back of the head. The equipment involved a Nexus device from which four channels were used to assess Electroencephalography (EEG), Electrocardiography (ECG), Electromyography (EMG), and Blood Volume Pulse (BVP) activations. The BVP sensor was placed on the index finger to measure changes in blood volume in the arteries and capillaries that are reflective of changes in heart rate and blood flow. The data were subjected to artifact analysis to remove spikes and random errors *via* an automatic feature of the device. Thus, segments with artifacts were excluded from further analyses.

#### Electromyography Artifact

An electromyogram analysis was conducted to assess EMG artifact, which is essentially electrical “noise” due to facial muscle activity in the proximity of the electrodes. The use of facial muscles, for example, when showing surprise or excitement, produces elevated EMG activity of the electrodes placed near these areas. In the present study, two electrodes were placed on the forehead above each eyebrow; thus, these EMG signals would be easy to capture. We evaluated facial muscle activation only given ample evidence that physical stress is reflected with enhanced movement of the facial muscle (e.g., Li et al., 2018; Casaccia et al., 2021).

#### Electrocardiography Alpha and Beta Waves

They were assessed using a single sensor placed at the Cz location. Signals were cleared of artifacts with segments with an enhanced error being deleted.

### Data Analyses

Data were analyzed using a multilevel means as the outcomes model, in which the within-person experimental conditions point estimates were contrasted using chi-square difference tests (Bryk and Raudenbush, 1992). Initially, an unconditional model was applied to the data to verify that ample levels of variance were within persons (over time) and between persons (across individuals) (Muthen and Satorra, 1995). The unconditional model for the BVP is as follows:

*Level-1 Model*
BVP_AMPij_ = β_0j_ + r_ij_
*Level-2 Model*
β_0j_ = γ_00_ + u_0j_

where, *i* denotes the number of observations (times) for *individual j*. The term β_0j_ is an intercept (mean for a dependent variable across time points for each of *j* individuals) of BVP, and r_*ij*_ is the residual term, suggesting that the levels of BVP in the *i* observations (times) vary across individuals *j*. The mean of BVP for each individual *j* (i.e., β_0j_) is then modeled as a function of the grand mean γ_00_ plus an error term u_0j_, suggesting that the levels in the dependent variable for each individual vary (variability across individuals associated with the unique effects of each person). The error terms r_*ij*_ and u_0j_ are expected to be uncorrelated and normally distributed, respectively. The structural model for comparing the experimental conditions is as follows:

*Level-1 Model*
BVP_AMPij_ = β_1_*Normative Outcome_*ij*_ + β_2_*Ability_ij_ + β_3_*Performance Avoidance_ij_
+ β_4_ × Personal Best_ij_ + β_5_ × Engagement_ij_ + r_ij_.
*Level-2 Model*
β*_1j_* = γ*_1_*+u*_1j_*
β*_2j_* = γ*_2_*+u*_2j_*
β*_3j_* = γ*_3_*+u*_3j_*
β*_4j_* = γ*_4_*+u*_4j_*
β*_5j_* = γ*_5_*+u*_5j_*

where, the intercept terms β_1_-β_5_ from level 1 are a function of their own gamma intercept plus an error term due to the person. Each of the two experimental conditions at a time was contrasted using chi-square difference tests. Prior to estimating multilevel models, certain model assumptions must be met. First, there must be ample levels of variability at each level in the analysis, which is evidenced by the estimation of the intra-class correlation (ICC) coefficient (Raudenbush and Bryk, 2002; Maas and Hox, 2005). The coefficient is estimated as the ratio of the between-level variance $\sigma_{u\,0}^{2}$ to that of the total variance (within $\sigma_{r}^{2}$ and between $\sigma_{u\,0}^{2}$) and makes use of the null model as follows (Kreft and de Leeuw, 2004):


(1) $$I\,C\,C=\frac{\sigma_{u\,0}^{2}}{\left(\sigma_{u\,0}^{2}+\sigma_{r}^{2}\right)}$$

where, $\sigma_{u\,0}^{2}$ is the between-person variance, and $\sigma_{r}^{2}$ is the within-person variability or variance in the dependent variable over time. The results indicated that for dependent variables BVP, EMG, alpha amplitude, and beta amplitude, the intraclass correlation coefficients (ICCs) were 0.832, 0.382, 0.513, and 0.396, respectively, suggesting the need to nest observations within persons. For additional prerequisites to conducing multilevel modeling with the present data see Supplementary Material.

## Results

### Vasoconstriction *via* Blood Volume Pulse

Table 2 summarizes the results of the means as an outcome multilevel model. As expected, the levels of blood volume pulse (BVP) were non-zero across all conditions. Chi-square difference tests suggested that there were significantly lower levels of BVP in the performance-avoidance goal condition than in the ability goal condition (χ^2^(1) = 15.271, *p* < .001), the personal best condition [χ^2^(1) = 10.146, *p* = .002], and the engagement goal condition [χ^2^(1) = 8.540, *p* = .004]. Thus, only the performance-avoidance goal condition was associated with vasoconstriction compared to the three above-mentioned conditions. All other effects were null. Interestingly, the levels of BVP observed during the outcome goal condition (grounded in normative comparisons) were not significantly different compared to the ability, personal best, and engagement goal conditions.

**TABLE 2:** Results from the multilevel model for the prediction of vasoconstriction, EMG artifact, and alpha and beta amplitude as a function of goal orientations.

Goal orientations	B (Mean)	S.E.	*T*-Test	*p*-value^†^
**Vasoconstriction**				
1. Normative outcome	12.333_a_	1.268	9.724	<0.001***
2. Ability	13.437_b,c_	1.174	11.45	<0.001***
3. Performance avoidance	12.392_c,b,d,e_	1.326	9.349	<0.001***
4. Personal best	13.351_d,c_	1.486	8.984	<0.001***
5. Engagement goal	13.224_e,c_	1.234	10.716	<0.001***
**EMG artifact**				
1. Normative outcome	9.271_a,b,d,e_	0.072	127.928	<0.001***
2. Ability	9.101_b,a_	0.154	58.926	<0.001***
3. Performance avoidance	9.143_c_	0.166	54.998	<0.001***
4. Personal best	9.101_d,a_	0.186	48.972	<0.001***
5. Engagement goal	9.107_e,a_	0.168	54.238	<0.001***
**Alpha Wave amplitude**				
1. Normative outcome	28.803_a_	1.312	21.959	<0.001***
2. Ability	28.722_b,e,d_	0.217	132.537	<0.001***
3. Performance avoidance	29.330_c,e_	0.259	113.327	<0.001***
4. Personal best	29.835_d,e,b_	0.263	113.274	<0.001***
5. Engagement goal	27.984_e,c,d,b_	0.202	138.821	<0.001***
**Beta wave amplitude**				
1. Normative outcome	27.765_a_	1.227	22.638	<0.001***
2. Ability	28.761_b_	0.264	108.755	<0.001***
3. Performance avoidance	28.148_c,e_	0.261	107.631	<0.001***
4. Personal best	28.757_d_	0.287	100.104	<0.001***
5. Engagement goal	29.320_e,c_	0.267	109.89	<0.001***

***p < .001. The model was run in the absence of the intercept term (means as outcomes multilevel model). Subscripts a through e refer to experimental manipulations 1 through 5. The presence of two or more subscripts indicates significant differences in outcome variables across the two conditions. ^†^Family wise error was adjusted using the Benjamini-Hochberg correction with a false discovery rate (FDR) equal to the level of significance **(i.**e., 5%). All significant effects remained after the FDR correction.

### Muscle Activation *via* Electromyographic Artifact

As shown in Table 2, non-zero EMG values were evident across all goal conditions. Between-goal comparisons indicated the presence of elevated muscular activity during the outcome goal condition (emphasizing normative evaluations), compared to the ability goal condition (χ^2^(1) = 4.407, *p* = .034), the personal best goal condition [χ^2^(1) = 4.322, *p* = .035], and the engagement goal condition [χ^2^(1) = 4.024, *p* = .042]. All other comparisons were null.

### Alpha and Beta Wave Amplitudes

As shown in Table 2, the lowest levels of alpha waves were observed during the engagement goal condition. Low levels of alpha waves are linked to enhanced concentration and focus; thus, lower scores represent positive outcomes. When contrasting goal conditions using chi-square difference tests, several significant findings emerged. Specifically, alpha waves were significantly lower during the engagement goal condition than in the performance-avoidance goal condition (χ^2^(1) = 16.819, *p* < .001), the personal best goal condition [χ^2^(1) = 31.127, *p* < .001], and ability goal condition [χ^2^(1) = 6.199, *p* = .012]. Lower alpha activation was also evidenced in the ability goal condition compared to the personal best goal condition [χ^2^(1) = 10.670, *p* = .001].

Beta waves, on the other hand, are adaptive when engaging in cognitive tasks, as they enhance alertness and concentration; thus, they can be viewed as having an inverse relationship to alpha waves. When contrasting point estimates across experimental conditions, the results indicated the presence of enhanced alertness (i.e., elevated scores in beta waves) during the engagement goal condition compared to the performance-avoidance goal condition [χ^2^(1) = 9.838, *p* = .002] only. No other comparisons exceeded the conventional levels of significance.

## Discussion

The present study attempted to evaluate the self-regulatory properties of achievement goal orientations following the extended theoretical protocol of Grant and Dweck (2003), separating performance goals with different foci, and after inducing a frustrating experience. Several interesting findings emerged and are discussed next.

The most important finding was that enhanced stress in the form of vasoconstriction and lack of concentration were evident in the performance-avoidance goal condition compared to most other conditions. Thus, the goal of avoiding failure was associated with an elevated stress response that can only be maladaptive for the successful completion of cognitive tasks. This finding agrees with previous results in that performance-avoidance goals induce negative affect, undermine motivation, and deplete energy resources (Roskes et al., 2014). Oertig et al. (2013) located the maladaptiveness of performance-avoidance goals to an ill-placed end state in which the emphasis is to move away from a target with no direction or goal. They further added that the resources involved in this goal pursuit are survival-based and cannot provide the “psychological nutriments necessary to thrive’ (p. 366). The present study adds to this line of thought, suggesting that during a performance-avoidance goal state, resources are scarce compared to other goal conditions. However, the present findings disagree with studies that favor avoidance goals for brief tasks that require attention to detail or involve urgent dangers (Koch et al., 2008; Ståhl et al., 2012).

A second important finding was that outcome goals that emphasized normative comparisons were the only mechanism associated with elevated muscular activity of the facial muscles during the task. This finding is important for several reasons. First, it validates the discrimination of performance pursuits from those having normative and non-normative evaluative criteria such as normative goals versus personal best goals, an important distinction in the works of Grant and Dweck (2003), Martin (2006), and Bong et al. (2013). Second, unnecessary facial activity such as frowns (for being unhapphy), a lowering of the brows (for being angry or frustrated), a raising of the eyebrows (to indicate surprise) [see Song et al. (2012), Cowen et al. (2021)] essentially consumes available resources that are necessary for effective cognitive regulation. Third, the switch from self-references (i.e., personal best) to normative goals was associated with enhanced facial reactivity and adds to the growing literature that suggests that the performance goals using self-referenced (Martin and Elliot, 2015) and external foci (Grant and Dweck, 2003) are valid, in that they can be distinguished from one another by empirical measures taken during performance.

Given the diverse foci of the different goal conditions and evidence on the engagement of different regulatory systems, the present study suggests that engagement goals are most adaptive and offer the least stressful experience compared to any of the performance (self or norm-referenced) approaches or avoidance goals. This finding has important implications for classroom teachers. For example, it is much more appropriate for the teacher to say, “let’s have fun and play with some math concepts” rather than say, “think faster than everybody else and solve more math problems compared to your classmates.” As evidenced in the present study, the two directions are likely associated with divergent pathways of self-regulation and may be linked to divergent physiological, emotional, and behavioral processes.

The present study has several limitations. First, the sample size was modest, allowing generality only to the population of college students from public universities. Non-random selection has contributed to this effect as a convenience sampling methodology was employed. However, the use of a within-person design in which each person became their own control increased our confidence in the validity of the experimental manipulations and the obtained differences. Second, the EEG measurements involved only four channels, and they may be amended to increase the error. Third, although a there was a relaxation session between primed goal conditions, there is no guarantee that carry-over effects are not present and even variable across persons, although we expect that random assignment of persons to conditions likely corrected any such biases. Third, we can only assume that participants were free from emotional problems and a disability as this inference was based on participants’ self-report. Nevertheless, the present study is one of the few that investigated physiological signals to enhance our understanding of the regulatory properties of goal orientations. To its advantage, experimentally induced goals account for some of the limitations of self-reports (Elliot and Murayama, 2008). In the future, it is suggested that additional objective means of evaluating the stress response, such as saliva tests to assess cortisol levels, are involved.

## Data Availability Statement

The raw data supporting the conclusions of this article will be made available by the authors, without undue reservation.

## Ethics Statement

The studies involving human participants were reviewed and approved by University of Crete Department of Psychology Ethics Committee. The patients/participants provided their written informed consent to participate in this study.

## Author Contributions

FA conceptualized the study and contributed to data analyses and the write-up of the manuscript. GA contributed to data analyses and the write-up of the quantitative sections. Both authors approved the final draft of the manuscript.

## Conflict of Interest

The authors declare that the research was conducted in the absence of any commercial or financial relationships that could be construed as a potential conflict of interest.

## Publisher’s Note

All claims expressed in this article are solely those of the authors and do not necessarily represent those of their affiliated organizations, or those of the publisher, the editors and the reviewers. Any product that may be evaluated in this article, or claim that may be made by its manufacturer, is not guaranteed or endorsed by the publisher.

## References

[B1] Abramson L. Y. Alloy L. B. Hankin B. L. Haeffel G. J. MacCoon D. G. Gibb B. E. (2002). “Cognitive vulnerability-stress models of depression in a self-regulatory and psychobiological context in,” in *Handbook of Depression*, eds Gotlib I. H. Hammen C. L. (New York, NY: Guilford Press), 268–294.

[B2] Asada H. H. Shaltis P. Reisner A. Rhee S. Hutchinson R. C. (2003). Mobile monitoring with wearable photoplethysmographic biosensors. *IEEE Eng. Med. Biol. Mag.* 22 28–40. 10.1109/memb.2003.1213624 12845817

[B3] Beilock S. L. Bertenthal B. I. McCoy A. M. Carr T. H. (2004). Haste does not always make waste: expertise, direction of attention, and speed versus accuracy in performing sensorimotor skills. *Psychon. Bull. Rev.* 11 373–379. 10.3758/bf03196585 15260208

[B4] Beilock S. L. Carr T. H. (2001). On the fragility of skilled performance: what governs choking under pressure? *J. Exp. Psychol. Gen.* 130 701–725. 10.1037/0096-3445.130.4.701 11757876

[B5] Beilock S. L. Carr T. H. (2005). When high-powered people fail: working memory and “choking under pressure” in math. *Psychol. Sci.* 16 101–105. 10.1111/j.0956-7976.2005.00789.x 15686575

[B6] Beilock S. L. Jellison W. A. Rydell R. J. McConnell A. R. Carr T. H. (2006). On the causal mechanisms of stereotype threat: can skills that don’t rely heavily on working memory still be threatened? *Pers. Soc. Psychol Bull.* 32 1059–1071. 10.1177/0146167206288489 16861310

[B7] Bong M. Woo Y. Shin J. (2013). Do students distinguish between different types of performance goals? *J. Exp. Educ.* 81 464–489. 10.1111/j.1365-2923.2012.04266.x 22626052

[B8] Bryk A. S. Raudenbush S. W. (1992). *Hierarchical Linear Models.* Newbury Park, CA: Sage.

[B9] Butler R. (1992). What young people want to know when: effects of mastery and ability goals on interest in different kinds of social comparisons. *J. Pers. Soc. Psychol.* 62 934–943. 10.1037/0022-3514.62.6.934

[B10] Butler R. (2006). Are mastery and ability goals both adaptive? evaluation, initial goal construction and the quality of task engagement. *Br. J. Educ. Psychol.* 76 595–611. 10.1348/000709905X52319 16953964

[B11] Carr T. H. (2015). Strengths and weaknesses of reflection as a guide to action: pressure assails performance in multiple ways. *Phenomenol. Cogn. Sci.* 14 227–252. 10.1007/s11097-014-9401-z

[B12] Casaccia S. Sirevaag E. Frank M. O’Sullivan J. Scalise L. Rohrbaugh W. (2021). Facial muscle activity: high-sensitivity noncontact measurement using laser doppler vibrometry. *IEEE Trans. Instrum. Meas.* 70 1–10. 10.1109/tim.2021.306056433776080

[B13] Chib V. S. De Martino B. Shimojo S. O’Doherty J. P. (2012). Neural mechanisms underlying paradoxical performance for monetary incentives are driven by loss aversion. *Neuron* 74 582–594. 10.1016/j.neuron.2012.02.038 22578508 PMC3437564

[B14] Cowen A. S. Keltner D. Schroff F. Jou B. Adam H. Prasad G. (2021). Sixteen facial expressions occur in similar contexts worldwide. *Nature* 589 251–257. 10.1038/s41586-020-3037-7 33328631

[B15] Dunning D. (2017). Normative goals and the regulation of social behavior: the case of respect. *Motiv. Emot.* 41 285–293. 10.1007/s11031-017-9616-8

[B16] Egloff B. Wilhelm F. H. Neubauer D. H. Mauss I. B. Gross J. J. (2002). Implicit anxiety measure predicts cardiovascular reactivity to an evaluated speaking task. *Emotion* 2 3–11. 10.1037/1528-3542.2.1.3 12899363

[B17] Elliot A. J. Church M. A. (1997). A hierarchical model of approach and avoidance achievement motivation. *J. Pers. Soc. Psychol.* 72 218–232. 10.1037//0022-3514.76.4.628 10234849

[B18] Elliot A. J. Murayama K. (2008). On the measurement of achievement goals: critique, illustration, and application. *J. Educ. Psychol.* 100 613–628. 10.1037/0022-0663.100.3.613

[B19] Ellis H. C. Ashbrook P. W. (1988). “Resource allocation model of the effects of depressed mood states on memory in,” in *Affect, Cognition, and Social Behavior*, eds Fiedler K. Forgas J. P. (Toronto: Hogrefe), 25–43. 10.1037//0096-3445.126.2.131

[B20] Fredricks J. A. Blumenfeld P. C. Paris A. (2004). School engagement: potential of the concept, state of the evidence. *Rev. Educ. Res.* 74 59–109. 10.3102/00346543074001059

[B21] Genov A. Shay I. Boone R. T. (2002). *Genov Modified Stroop Task [Online].* Available online at: http://facpub.stjohns.edu/~booner/GmstSite/index.htm (accessed February 14, 2002).

[B22] Grant H. Dweck C. S. (2003). Clarifying achievement goals and their impact. *J. Pers. Soc. Psychol.* 85 541–553. 10.1037/0022-3514.85.3.541 14498789

[B23] Hill D. M. Shaw G. (2013). A qualitative examination of choking under pressure in team sport. *Psychol.Sport Exerc.* 14 103–110. 10.1016/j.psychsport.2012.07.008

[B24] Izzo J. L. Shykoff B. E. (2001). Arterial stiffness: clinical relevance, measurement, and treatment. *Rev. Cardiovasc. Med.* 34 37–40. 12478235

[B25] Kirschfeld K. (2005). The physical basis of alpha waves in the electroencephalogram and the origin of the “Berger effect”. *Biol. Cybern.* 92 177–185. 10.1007/s00422-005-0547-1 15739111

[B26] Kleiger R. E. Miller J. P. Bigger J. T. Moss A. J. (1987). Decreased heart rate variability and its association with increased mortality after acute myocardial infarction. *Am. J. Cardiol.* 59 256–262. 10.1016/0002-9149(87)90795-8 3812275

[B27] Koch S. Holland R. W. van Knippenberg A. (2008). Regulating cognitive control through approach-avoidance motor actions. *Cognition* 109 133–142. 10.1016/j.cognition.2008.07.014 18835601

[B28] Kreft I. G. G. de Leeuw J. (2004). *Introducing multilevel modeling.* Thousand Oaks, CA: Sage.

[B29] Li X. Hong K. Liu G. (2018). Detection of physical stress using facial muscle activity. *J. Optical Technol.* 85 562–569. 10.1364/jot.85.000562

[B30] Liem G. Ginns P. Martin A. Stone B. Herrett M. (2012). Personal best goals and academic and social functioning: a longitudinal perspective. *Learn. Instruction* 22 222–230. 10.1016/j.learninstruc.2011.11.003

[B31] Luijcks R. Hermens H. J. Bodar L. Vossen C. J. van Os J. Lousberg R. (2014). Experimentally induced stress validated by EMG activity. *PLoS One* 9:e95215. 10.1371/journal.pone.0095215 24736740 PMC3988146

[B32] Lundberg U. Kadefors R. Melin B. Palmerud G. Hassmen P. Engstrom M. (1994). Psychophysiological stress and EMG activity of the trapezius muscle. *Int. J. Behav. Med.* 1 354–370. 10.1207/s15327558ijbm0104_5 16250795

[B33] Maas C. J. M. Hox J. J. (2005). Sufficient sample sizes for multilevel modeling. *Methodology* 1 86–92. 10.1027/1614-2241.1.3.86

[B34] Martin A. J. (2006). Personal bests (PBs): a proposed multidimensional model and empiricalanalysis. *Br. J. Educ. Psychol.* 76 803–825. 10.1348/000709905X55389 17094887

[B35] Martin A. J. Elliot A. J. (2015). The role of personal best (PB) goal setting in students’ academic achievement gains. *Learn. Individ. Diff.* 45 222–227. 10.1016/j.lindif.2015.12.014

[B36] Mesagno C. Harvey J. T. Janelle C. M. (2011). Self-presentation origins of choking: evidence from separate manipulations of pressure. *J.Sport Exerc. Psychol.* 33 441–459. 10.1123/jsep.33.3.441 21659672

[B37] Mesagno C. Hill D. M. (2013). Choking under pressure debate: is there chaos in the brickyard? *Int. J.Sport Psychol.* 44 288–293.

[B38] Muthen B. O. Satorra A. (1995). Complex sample data in structural equation modeling. *Sociol. Methodol.* 25 267–316. 10.2307/271070

[B39] Oertig D. Schüler J. Schnelle J. Brandstätter V. Roskes M. Elliot A. J. (2013). Avoidance goal pursuit depletes self-regulatory resources. *J. Pers.* 81 365–375. 10.1111/jopy.12019 23126507

[B40] Papantoniou G. Moraitou D. Kaldrimidou M. Plakitsi K. Filippidou D. Katsadima E. (2012). Affect and cognitive interference: an examination of their effect on self-regulated learning. *Educ. Res. Int.* 2012:579590.

[B41] Patron E. Messerotti Benvenuti S. Favretto G. Valfrè C. Bonfà C. Gasparotto R. (2012). Association between depression and heart rate variability in patients after cardiac surgery: a pilot study. *J. Psychosom. Res.* 73 42–46. 10.1016/j.jpsychores.2012.04.013 22691558

[B42] Peeper E. Harvey R. Lin I. M. Tylova H. Moss D. (2007). Is there more to blood volume pulse than heart rate variability, respiratory sinus arrhythmia, and cardiorespiratory synchrony? *Biofeedback* 35 54–61.

[B43] Pekrun R. Elliot A. J. Maier M. A. (2009). Achievement goals and achievement emotions: testing a model of their joint relations with academic performance. *J. Educ. Psychol.* 101 115–135. 10.1037/a0013383

[B44] Raudenbush S. W. Bryk A. S. (2002). *Hierarchical Linear Models.* Newbury Park, CA: Sage.

[B45] Reeve J. Jang H. Carrell D. Jeon S. Barch J. (2004). Enhancing students’ engagement by increasing teachers’ autonomy support. *Motiv. Emot.* 28 147–169. 10.1023/b:moem.0000032312.95499.6f

[B46] Reinhardt T. Schmahl C. Wüst S. Bohus M. (2012). Salivary cortisol, heart rate, electrodermal activity and subjective stress responses to the mannheim multicomponent stress test (MMST). *Psychiatry Res.* 198 106–111. 10.1016/j.psychres.2011.12.009 22397919

[B47] Rissén D. Melin B. Sandsjö L. Dohns I. Lundberg U. (2002). Psychophysiological stress reactions, trapezius muscle activity, and neck and shoulder pain among female cashiers before and after introduction of job rotation. *Work Stress* 16 127–137. 10.1080/02678370210141530

[B48] Roskes M. Elliot A. J. De Dreu C. K. W. (2014). Why is avoidance motivation problematic, and what can be done about it? *Curr. Dir. Psychol. Sci.* 23 133–138. 10.1177/0963721414524224

[B49] Sarason I. G. Pierce G. R. Sarason B. R. (eds) (1996). *Cognitive Interference: Theories, Methods, and Findings.* Mahwah, N.J: Lawrence Erlbaum Publishers.

[B50] Song J. Wang L. Wang W. (2012). “Eyebrow segmentation based on binary edge image,” in *Intelligent Computing Technology. Lecture Notes in Computer Science*, eds Huang D.-S. Jiang C. Bevilacqua V. Figueroa J. C. (New York, NY: Springer), 350–356. 10.1007/978-3-642-31588-6_45

[B51] Ståhl T. Van Laar C. Ellemers N. (2012). The role of prevention focus under stereotype threat: initial cognitive mobilization is followed by depletion. *J. Pers. Soc. Psychol.* 102 1239–1251. 10.1037/a0027678 22409487

[B52] Taylor C. B. (2010). Depression, heart rate related variables and cardiovascular disease. *Int. J. Psychophysiol.* 78 80–88. 10.1016/j.ijpsycho.2010.04.006 20420861

[B53] Wahlström J. Lindegård A. Ahlborg G. Ekman A. Hagberg M. (2003). Perceived muscular tension, emotional stress, psychological demands and physical load during VDU work. *Int. Arch. Occup. Environ. Health* 76 584–590. 10.1007/s00420-003-0454-5 12898271

