# Supplementary material for: The Stressful Experience of Goal Orientations Under Frustration: Evidence Using Physiological Means
Source: Front Psychol. 2022 Apr 13;13:823655. doi: 10.3389/fpsyg.2022.823655 (PMC9043329; doi:10.3389/fpsyg.2022.823655)
Supplement: Supplementary file 1 [file Data_Sheet_1.docx]

**Supplementary Material**

### **Prerequisites To Modeling Nested Data**

An important concept that contributes to the decision to employ multilevel modeling techniques is the “design effect” index (Muthen and Satorra, 1995). The design effect targets the correction of the negative bias associated with nested data owing to the violation of the independence of standard errors. It contributes a multiplier that corrects the standard errors. This is computed as follows:

Design Effect = 1 + (n_c_-1)*ICC (Equation 1)

where n_c_ is the number of level-1 units that comprise the clustering variable. As shown in the above equation, the design effect is a function of both the number of units in the clustering variable and the magnitude of the ICC.

Another important consideration relates to the variability and differentiation of the level-1 units (termed “reliability” in multilevel models) and how reliably these ordinary least squares estimates can discriminate level-2 units. Estimates below .20 suggest that a random coefficient may need to be considered to be fixed in subsequent analyses.

A last consideration relates to testing for the homogeneity of error variances at level-1. If the level-1 variance estimates do not vary randomly over the level-2 units, biased standard errors can emerge (Raudenbush and Bryk, 2002). In the present study, we tested for the presence of homogeneous variances at level-1 predictors, that is, the time-series variables using the following log-linear model:

$\sigma_{ij}^{2}=exp\{a_{0}+a_{1}{Time}_{ij}\}$ (Equation 2)

If level-1 variances were not homogeneous, a heterogeneous within-person variance model was estimated using full maximum likelihood. The fifth and last consideration relates to knowing the distribution of the outcome variable and controlling for deviations from expectations (i.e., the normal curve). To this end, robust standard errors were utilized to account for possible dependent variable distribution misspecifications (Raudenbush and Bryk, 2002). All analyses were conducted using the HLM 7 software (Bryk and Raudenbush, 1992), and the alpha level was set at 5% for a two-tailed test.

Results indicated design effect (DEFF) estimates across BVP, EMG, alpha, and beta amplitudes of 26.794, 12.847, 16.899, and 13.277, respectively, suggesting the need to employ a nested structure in the data analysis strategy. Furthermore, following the baseline model, homogeneity of the variances of the level-1 units was assessed and based on the deviance statistic, the assumption was not met for all dependent variables across experimental conditions. Thus, the model estimation involved allowing for heterogeneous variances σ^2^ (see Supplemental Figure 1 below for between-person variability in BVP). Finally, reliability estimates that are indicative of between-person separation were 0.998, 0.994, 0.997, and 0.995 for BVP, EMG, alpha, and beta amplitudes, respectively. Thus, collectively, all the information proposed the need to accommodate the nested structure of the present data.

**Supplemental Figure 1**

Boxplot showing variability between persons in their BVP levels. Visual and statistical

analyses confirmed the need to model heterogeneous variances.

Level-1 Units
